# Effects of neurally adjusted ventilatory assist on air distribution and dead space in patients with acute exacerbation of chronic obstructive pulmonary disease

**DOI:** 10.1186/s13054-017-1714-1

**Published:** 2017-06-02

**Authors:** Qin Sun, Ling Liu, Chun Pan, Zhanqi Zhao, Jingyuan Xu, Airan Liu, Haibo Qiu

**Affiliations:** 1 Department of Critical Care Medicine, Zhongda Hospital, School of Medicine, Southeast University, No.87, Dingjiaqiao Road, Gulou District, Nanjing, 210009 Jiangsu China; 2 Institute of Technical Medicine, Furtwangen University, Villingen-Schwenningen, Germany

**Keywords:** Intratidal gas distribution, Vd/Vt, Patient-ventilator interaction, Work of trigger, Electrical impedance tomography

## Abstract

**Background:**

Neurally adjusted ventilatory assist (NAVA) could improve patient-ventilator interaction; its effects on ventilation distribution and dead space are still unknown. The aim of this study was to evaluate the effects of varying levels of assist during NAVA and pressure support ventilation (PSV) on ventilation distribution and dead space in patients with acute exacerbation of chronic obstructive pulmonary disease (AECOPD).

**Methods:**

Fifteen mechanically ventilated patients with AECOPD were included in the study. The initial PSV levels were set to 10 cmH_2_O for 10 min. Thereafter, the ventilator mode was changed to NAVA for another 10 min with the same electrical activity of the diaphragm as during PSV. Furthermore, the ventilation mode was switched between PSV and NAVA every 10 min in the following order: PSV 5 cmH_2_O; NAVA 50%; PSV 15 cmH_2_O; and NAVA 150% (relative to the initial NAVA support level). Ventilation distribution in the lung was evaluated in percentages in regions of interest (ROI) of four anteroposterior segments of equal height (ROI1 to ROI4 represents ventral, mid-ventral, mid-dorsal, and dorsal, respectively). Blood gases, ventilation distribution (electrical impedance tomography), diaphragm activity (B-mode ultrasonography), and dead space fraction (PeCO_2_ and PaCO_2_) were measured.

**Results:**

The trigger and cycle delays were lower during NAVA than during PSV. The work of trigger was significantly lower during NAVA compared to PSV. The diaphragm activities based on ultrasonography were higher during NAVA compared to the same support level during PSV. The ventilation distribution in ROI4 increased significantly (*P* < 0.05) during NAVA compared to PSV (except for a support level of 50%). Similar results were found in ROI3 + 4. NAVA reduced dead space fraction compared to the corresponding support level of PSV.

**Conclusions:**

NAVA was superior to PSV in AECOPD for increasing ventilation distribution in ROI4 and reducing dead space.

**Trial registration:**

Clinicaltrials.gov, NCT02289573. Registered on 12 November 2014.

## Background

The acute exacerbation of chronic obstructive pulmonary disease (AECOPD) is a disease state characterized by expiratory airflow limitation, increasing dead space, and diaphragm dysfunction [1], and invasive mechanical ventilation (MV) is required in the presence of severe respiratory failure [2]. Earlier studies have found less ventilation distribution in the near-diaphragm region and more dead space in patients with chronic obstructive pulmonary disease (COPD) [3, 4]. Improving heterogeneous ventilation distribution and decreasing dead space are important during MV in AECOPD patients [3, 4].

Pressure support ventilation (PSV), an MV mode with constant pressure assistance, is commonly used in patients with AECOPD; however, PSV has been proven to intensify heterogeneous ventilation distribution in patients with acute lung injury (ALI) [5]. Neurally adjusted ventilatory assist (NAVA) is a new MV mode that is triggered using diaphragm electrical activity (EAdi) and gives the amount of pressure assistance that is proportional in relation to EAdi. In theory, NAVA can improve heterogeneous ventilation distribution by decreasing the contraction of diaphragm, which Paul Blankman and his colleagues have proven [5].

Patients with AECOPD also suffer from increasing dead space and decreasing ventilation efficiency [4]. Over-assistance during PSV may exacerbate this problem [6]. NAVA provides the amount of pressure assistance in relation to EAdi and reduces the risk of over-assistance due to downregulation of the EAdi signal [7–13], which may be a solution to increasing dead space during MV in patients with AECOPD.

Based on this analysis, we designed a study to distinguish the difference between NAVA and PSV on ventilation distribution and dead space in patients with AECOPD. We hypothesized that NAVA could be a better MV mode for patients with AECOPD by improving heterogeneous ventilation distribution and decreasing dead space.

## Methods

### Study population

All study patients or their family members signed informed consent forms. The protocol was approved by the local institutional human investigations committee (IEC for Clinical Research of Zhongda Hospital, Affiliated to Southeast University, 2014ZDSYLL086.0). Patients who were admitted to the intensive care unit (ICU), intubated because of AECOPD, and ventilated by PSV were eligible for inclusion in the study. The exclusion criteria were as follows: (1) age <18 years or >85 years; (2) ventilated because of oesophageal surgery in the previous 12 months; (3) oesophageal bleeding in the previous 30 days; (4) history of oesophageal varices; (5) facial trauma and surgery; (6) haemodynamic instability despite adequate filling (i.e. the need for continuous infusion of epinephrine or vasopressin, or dopamine >5 μg/kg/min or norepinephrine >0.1 μg/kg/min to maintain systolic arterial blood pressure >90 mmHg); (7) coagulation disorders (international normalised ratio (INR) >1.5 and partial thromboplastin time (PTT) >44 s); and (8) inclusion in other research protocols.

### Experimental protocol

At the time of enrolment, all patients were mechanically ventilated using PSV and Servo-I (Maquet, Solna, Sweden). The standard nasogastric tubes of all included patients were replaced by the modified NAVA tube positioned according to the manufacturer’s recommendations [7]. Airway suctioning was performed adequately before the beginning of the protocol. There was no airway suctioning during the protocol. During the entire recording period, positive end-expiratory pressure (PEEP), fraction of inspired oxygen (FiO_2_), inspiratory trigger, and cycling-off settings were maintained as set by the clinician in charge of the patient. The initial PSV levels were set to 10 cmH_2_O (denoted as PSV 100) for 10 min. Thereafter, the ventilator mode was changed to NAVA for another 10 min. The EAdi during PSV 100 was used to titrate to the corresponding initial NAVA gain (NAVA100). Furthermore, the ventilation mode switched between PSV and NAVA every 10 min in the following order: PSV of 5 cmH_2_O (PSV50); NAVA 50% support level of NAVA100 (NAVA50); PSV of 15 cmH_2_O (PSV150); and NAVA 150% support level of NAVA100 (NAVA150).

The electrical impedance tomography (EIT) measurements were performed using a 16-electrode silicon belt placed around the patient’s thoracic cage just below the nipples and between the 4th and 5th intercostal spaces (Pulmovista 500, Dräger Medical, Lübeck, Germany). The data were gathered at a sampling frequency of 20 Hz. Ventilation distribution was evaluated in percentages in regions of interest (ROI) of four anteroposterior segments of equal height [14]. ROI1 corresponded to the most non-dependent regions, whereas ROI4 denoted the most dependent regions. The regional ventilation-delay index estimating the early and late opening of lung tissues was calculated according to a previous study [15, 16]. The ventilator waveform was recorded using a Servo-I ventilator, and all signals were digitized at 100 Hz and were stored for offline analysis (NeuroVent Research Inc., Toronto, ON, Canada).

Two minutes before changing the ventilator mode, the diaphragm activity was measured using B-mode ultrasonography (FUJiFILM SonoSite Inc.). The ultrasound images of the right hemidiaphragm were obtained using a mechanical sector scanner fitted with a 5-MHz transducer (Honeywell Ultraimager). The transducer was placed below the right costal margin and, with the liver providing an acoustic window, saggital views of the posterior portion of the diaphragm were obtained until a maximum diaphragmatic excursion was observed. The transducer was held at the described position and the movement of the right hemidiaphragm was recorded as an M-mode tracing. Five consecutive respiratory cycles were measured, and the average movement was recorded as diaphragm activity.

The dead space fraction (Vd/Vt) was estimated based on the following equation: Vd/Vt = (PaCO_2_ – PeCO_2_)/PaCO_2_,

where PaCO_2_ is obtained from the arterial blood ventilation measurements and PeCO_2_ is the mixed expired PCO_2_ [17]. A Douglas bag was attached to the exhalation port of the ventilator, and the PeCO_2_ was collected over 15 min. At the end of the timed collection, PeCO_2_ of the ventilation collected in the Douglas bag was analysed using a blood ventilation analyser (Radiometer, Westlake, OH, USA). PaO_2_ and PaCO_2_ were measured after each step.

### Statistics

Before starting the protocol, we calculated the sample size using the following formula: *n* = 2 × ((α + β)σ/δ)^2^, and the minimum sample size was 13. The statistical analyses were performed using SPSS 20 (Chicago, IL, USA). The values are shown as the mean ± SD unless specified otherwise. For the normally distributed data, paired samples *t* tests and Bonferroni correction were used. We analysed the data that did not meet the assumption of normality using the means of non-parametric statistical tests. The correlation of diaphragm activity and ventilation distribution in ROI 4 was evaluated using Pearson two-tailed test. The differences were considered to be significant when *P* < 0.05.

The primary endpoint of the study was to investigate changes in ventilation distribution and Vd/Vt between NAVA and PSV. Our secondary endpoints included diaphragm activity, regional compliance, and regional ventilation delay. Because ventilation distribution data and regional ventilation delay did not meet the assumption of normality, we analysed them using non-parametric statistical tests. In this study, we also investigated the changes in ventilation distribution in gravity-dependent lung regions, dead space fraction between NAVA and PSV and diaphragm activity. Additionally, we analysed the differences in EAdi, peak pressure, respiratory rate, mean inspiratory pressure, expiratory tidal volume (Vt), volume, trigger delay (in seconds), cycle delay (in seconds), work of breathing (in μV∙s) and work of trigger (in μV∙s) between PSV and NAVA. For both PSV 100 and NAVA 100, there were no significant differences between peak EAdi.

## Results

Fifteen patients with AECOPD were admitted to this study from January 2015 to February 2016. The entry characteristics of the study population are presented in Table 1.

**Table 1:** Main characteristics of patients at inclusion

Patient	Sex	Age (years)	APACHE II	SOFA	Cause of AECOPD	PEEPi (cmH_2_O)	Ventilation time (days)
1	M	85	15	10	Sepsis	6	4
2	M	79	10	5	Sepsis	4	9
3	F	67	12	6	Sepsis	3	5
4	M	85	15	12	Sepsis	8	15
5	M	83	14	12	Sepsis	3.6	19
6	M	74	15	11	Sepsis	4	16
7	M	85	25	9	Sepsis	4	5
8	M	79	24	6	Sepsis	4	5
9	M	84	23	10	Sepsis	4.2	5
10	M	81	24	12	Sepsis	5	6
11	M	73	18	7	Sepsis	3.6	3
12	M	76	13	3	Sepsis	4	10
13	F	62	21	9	Sepsis	3	10
14	M	82	24	12	Sepsis	2	3
15	M	84	23	10	Sepsis	4.2	4
Mean ± SD		78.6 ± 7.01	18.4 ± 5.22	8.93 ± 2.89		4.17 ± 1.39	7.93 ± 5.11

*AECOPD* acute exacerbation of chronic obstructive pulmonary disease, *APACHE* Acute Physiology and Chronic Health Evaluation, *F* Female, *M* Male, *PEEP* positive end-expiratory pressure, *SOFA* Sequential Organ Failure Assessment

The ventilatory data, including the levels of pressure support and the resulting EAdi values during each PSV and NAVA step, are shown in Table 2. There was no difference in assist pressure or tidal volume between PSV50 and NAVA50 (Table 2). By increasing the level of assist, tidal volume and mean inspiratory pressure increased, whereas the respiratory rate decreased during PSV and NAVA (Table 2). At the highest applied ventilatory assist level, tidal volume was significantly higher with PSV compared to NAVA (Table 2). At different support levels, trigger delay and cycle delay were lower during NAVA than during PSV.

**Table 2:** Respiratory parameters varying pressure support and NAVA assist levels

Ventilator mode	50%		100%		150%	
	PSV	NAVA	PSV	NAVA	PSV	NAVA
Assist pressure (cmH_2_O)	6.73 ± 0.70	6.88 ± 1.08	8.20 ± 0.73^b^	7.68 ± 1.39	9.74 ± 1.23^c^	7.94 ± 1.25
Ppeak (cmH_2_O)	11.35 ± 0.91	12.88 ± 3.30	16.01 ± 0.84	16.52 ± 4.82	20.77 ± 0.94	18.81 ± 4.29
EAdi (μV)	14.69 ± 8.72	11.67 ± 8.64	11.48 ± 7.75	11.43 ± 8.13	14.33 ± 6.87^c^	11.86 ± 6.34
Respiratory rate (/min)	22.58 ± 5.28	22.34 ± 4.58	21.00 ± 7.71	23.06 ± 5.40	18.10 ± 6.67	19.59 ± 5.52
MVe (L/min)	7.66 ± 2.58	7.39 ± 2.20	8.58 ± 4.22	8.41 ± 3.32	8.84 ± 3.48^c^	7.65 ± 3.00
Inspiratory time (s)	0.93 ± 0.21	1.02 ± 0.14	1.10 ± 0.45	1.01 ± 0.21	1.22 ± 0.55	1.05 ± 0.18
Vt (L)	0.35 ± 0.09	0.34 ± 0.10	0.42 ± 0.14	0.38 ± 0.14	0.53 ± 0.21^c^	0.40 ± 0.11
Trigger delay (s)	0.17 ± 0.08^a^	0.05 ± 0.02	0.25 ± 0.23^b^	0.06 ± 0.06	0.34 ± 0.18^c^	0.06 ± 0.05
Cycle delay (s)	0.11 ± 0.10^a^	0.02 ± 0.01	0.32 ± 0.49^b^	0.06 ± 0.11	0.48 ± 0.72^c^	0.05 ± 0.07
Work of breathing (μV∙s)	7.31 ± 5.76	6.20 ± 5.96	6.10 ± 5.83	5.11 ± 2.31	7.33 ± 4.13	6.26 ± 3.91
Work of trigger (μV∙s)	1.20 ± 0.68^a^	0.89 ± 0.36	1.52 ± 1.25^b^	1.11 ± 0.59	2.17 ± 1.08^c^	1.20 ± 0.92
(ROI1 + 2) × Vt (L/min)	0.20 ± 0.16^a^	0.17 ± 0.13	0.26 ± 0.15	0.20 ± 0.14	0.26 ± 013^c^	0.21 ± 0.14
(ROI3 + 4) × Vt (L/min)	0.21 ± 0.12	0.19 ± 0.09	0.20 ± 0.12	0.20 ± 0.11	0.24 ± 0.12	0.22 ± 0.10

*EAdi* electrical activity of the diaphragm, *MVe* minute ventilatory volume, *NAVA* neutrally-adjusted ventilator assist, *Ppeak* peak pressure, *PSV* pressure support ventilation, *ROI* regions of interest, *Vt* tidal volume

^a^Significant different versus NAVA 50%

^b^Significant different versus NAVA 100%

^c^Significant different versus NAVA 150%

The work of trigger was significantly lower during NAVA compared to PSV at three support levels, but there was no difference in the work of breathing (Table 2).

**Fig. 1:**
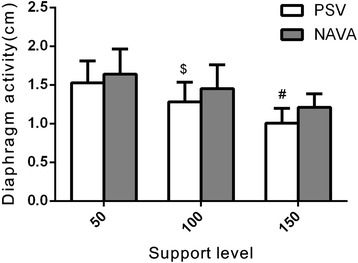
Diaphragm activity in both PSV and NAVA. The *bars* represent the mean value and the *lines* represent the standard deviation. ^$^Significant differences versus NAVA 100%; ^#^significant differences versus NAVA 150%. *NAVA* neutrally-adjusted ventilator assist, *PSV* pressure support ventilation

The diaphragm activity in patients with AECOPD decreased when the support level increased for both PSV and NAVA (Fig. 1). However, the diaphragm activities were higher during NAVA compared to the same support level during PSV (Fig. 1).

**Fig. 2:**
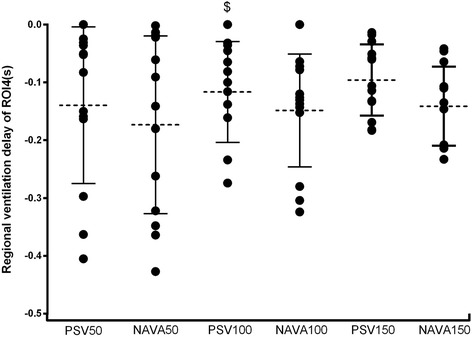
Regional ventilation delay of ROI4 of every patient during a different ventilation mode. The *dotted lines* represent the medians and the *solid lines* represent quartiles. The negative value means that the ventilation began earlier than the null point. ^$^Significant differences versus NAVA 100%. *NAVA* neutrally-adjusted ventilator assist, *PSV* pressure support ventilation, *ROI* regions of interest

The ventilation distribution in ROI4 increased significantly (*P* < 0.05) during NAVA compared to PSV (except for a support level of 50%). Similar results were found in ROI3 + 4. The ventilation distribution decreased in the ROI1 and ROI2 regions during NAVA (Table 3). The regional ventilation delay also varied in different lung regions. Four ROI had different ventilation times. We found that in the ROI4 region inspiration began earlier in NAVA compared to PSV (Fig. 2).

**Table 3:** Gas distribution in four regions for both NAVA and PSV

	50%			100%			150%		
	PSV	NAVA	*P*	PSV	NAVA	*P*	PSV	NAVA	*P*
ROI 1	11.98 ± 11.17	11.76 ± 10.65	0.715	12.89 ± 8.79	11.86 ± 8.89	0.057	12.21 ± 8.49	11.11 ± 9.37	0.039^a^
ROI 2	38.60 ± 17.65	36.36 ± 18.71	0.054	44.26 ± 13.63	39.02 ± 15.07	0.003^a^	41.94 ± 14.80	37.58 ± 13.87	0.004^a^
ROI 3	39.79 ± 21.54	41.37 ± 22.41	0.220	35.75 ± 18.50	40.13 ± 19.64	0.014^a^	37.58 ± 18.82	41.37 ± 18.82	0.18
ROI 4	9.66 ± 8.42	10.98 ± 9.05	0.158	7.53 ± 4.98	9.25 ± 5.54	0.005^a^	9.10 ± 6.71	10.89 ± 8.06	0.020^a^
ROI3 + 4	49.46 ± 27.28	52.35 ± 27.97	0.093	43.28 ± 20.22	49.38 ± 22.00	0.007^a^	46.68 ± 21.94	52.26 ± 22.22	0.004^a^

*NAVA* neutrally-adjusted ventilator assist, *PSV* pressure support ventilation, *ROI* regions of interest

^a^Significantly different versus NAVA

**Fig. 3:**
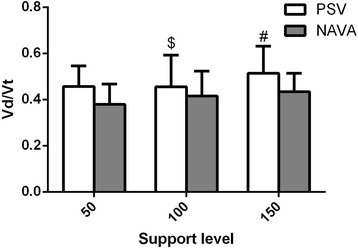
Vd/Vt in both PSV and NAVA. The *bars* represent the mean values and the *lines* represent the standard deviation. ^$^Significant differences versus NAVA 100%; ^#^significant differences versus NAVA 150%. *NAVA* neutrally-adjusted ventilator assist, *PSV* pressure support ventilation

**Fig. 4:**
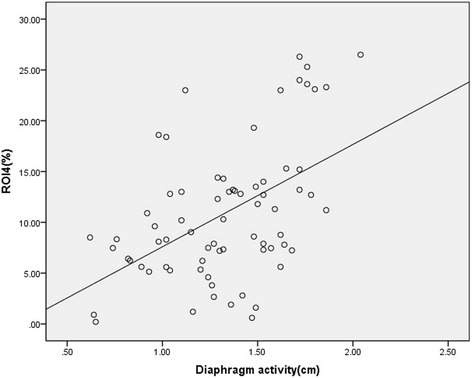
Correlation between the diaphragm activity and ventilation distribution in ROI4. *R* ^2^ = 0.56, *P* < 0.01. *ROI* regions of interest

Higher levels of assist resulted in higher Vd/Vt values, and NAVA reduced Vd/Vt values compared to the corresponding support level of PSV (Fig. 3). In this study, we also analysed the consistency of diaphragm activity and ventilation distribution in ROI4 and found that the correlation coefficient *R* ^2^ was 0.251 (*P* < 0.01) (Fig. 4).

## Discussion

In this study, we found the following: (1) ventilation distribution in ROI4 increased significantly during NAVA compared to PSV (except for a support level of 50%), and similar results were found in ROI3 + 4; (2) diaphragm activities measured using ultrasonography were higher during NAVA compared to the same support level during PSV; and (3) NAVA reduced Vd/Vt values compared to the corresponding support level of PSV. Additionally, we found that, at different support levels, trigger delay and cycle delay were lower during NAVA than during PSV in patients with AECOPD. The work of trigger was significantly lower during NAVA compared to PSV at three support levels.

During NAVA, EAdi, an expression of the respiratory centre’s activity, was used to trigger and cycle the ventilator, rather than a pneumatic signal located at the airway opening or inside the ventilator, which can significantly reduce trigger delay and cycle delay. Previous studies suggested that NAVA also decreased the work of trigger compared to PSV [18, 19]. In PSV, the ventilator settings required a remarkable inspiratory effort to minimize the miss-triggering. In contrast with NAVA (when the ventilator’s delivered cycle was triggered by the EAdi), as the ventilator’s delivered pressurization immediately followed the increase in EAdi signal (without any depression on the pressure-time curve), less inspiratory effort was theoretically needed to trigger the ventilator. In our results, the work of trigger in NAVA was significantly lower than in PSV, and these results were consistent with findings in previous research. No significant differences were found in the work of breathing between NAVA and PSV.

Lowhagen and colleagues introduced the intertidal ventilation distribution based on EIT measurements during a trial consisting of ten patients with ALI [20]. They used the intertidal ventilation distribution to analyse how the tidal volume was distributed within the lung and found that the ventilation distribution in the ROI4 region increased during NAVA compared to PSV. In the present study, we found the same phenomenon in patients with AECOPD.

Many cross-sectional studies with careful matching of patients with controls have revealed the complexity of diaphragm dysfunction in COPD [21, 22]. Jérôme Cecchini and his colleagues found, that in critically ill and mechanically ventilated patients, increasing levels of PSV and NAVA reduced the diaphragm activity [23]. However, compared with PSV, NAVA resulted in a predominant contribution of the diaphragm to inspiratory effort. Our results showed a higher diaphragm activity during NAVA compared to PSV, and we found a correlation between diaphragm activity and ventilation distribution in ROI4. Furthermore, we found that in ROI4 inspiration began earlier in NAVA compared to PSV, which indicated an early onset of diaphragm contraction and a better patient-ventilator synchronisation with NAVA. Based on these results, we insist that NAVA could increase ventilation distribution in ROI4 by improving the diaphragm activity.

Dead space plays an important role in ventilation efficiency in patients with AECOPD, but it is difficult to measure directly. Therefore, we measured Vd/Vt instead of dead space. Vd/Vt is a vital respiratory physiological index that represents the efficiency of lung ventilation [23]. We found that, in patients with AECOPD, Vd/Vt decreased significantly during NAVA. To explain this finding, we compared the tidal volume between PSV and NAVA and found that during NAVA the tidal volume was comparable despite the different levels of assist, whereas the tidal volume increased during increasing levels of PSV. This can be explained by the downregulation of the EAdi signal at higher assist levels during NAVA [7]. Additionally, we calculated the tidal volume distributed in ROI and 2 ((ROI 1 + 2) × Vt) and found that during NAVA the tidal volume distributed in ROI 1 and 2 ((ROI 1 + 2) × Vt) decreased significantly when compared to the same support level of PSV. This result meant that there was less ventilation distribution in the non-dependent lung region where pulmonary hyperinflation occurred easily. Improved ventilation distribution was another factor in the decreased Vd/Vt during NAVA.

There were several limitations in this study that must be addressed. One limitation of the study design was that the order of ventilation modes was not randomized. The results might be biased from the previous ventilator settings. Furthermore, the period of each ventilator setting was brief (10 min), and there could be a risk that some physiologic variables could not reach their steady states within that period. The reason for those insignificant differences might be clarified in the findings between NAVA and PSV. Nevertheless, we identified significant differences in ventilation distribution, work of trigger, and Vd/Vt values, which could be even more significant given a longer period of different settings. Even if the level of assistance in NAVA has been determined according to the usual procedure described by the manufacturer and reported in previous studies (NAVA gain or proportionality factor between EAdi and pressure delivered by the ventilator, set to obtain the same peak pressure in NAVA as in PSV), the comparability of assistance levels in PSV and NAVA is questionable. Other limitations included a one-centre design and small size. Further studies are needed.

## Conclusions

NAVA can increase ventilation distribution in the near-dorsal lung region and decrease Vd/Vt value in patients with AECOPD. Furthermore, NAVA improves the patient-ventilator interaction in intubated patients with AECOPD and decreases the work of trigger. NAVA can be beneficial in this patient population. Additional studies should be conducted to determine whether NAVA could decrease ventilation time and improve outcome.

## Abbreviations

AECOPD: Acute exacerbations of chronic obstructive pulmonary disease
ALI: Acute lung injury
COPD: Chronic obstructive pulmonary disease
EAdi: Diaphragm electrical activity
EIT: Electrical impedance tomography
MV: Mechanical ventilation
NAVA: Neurally adjusted ventilatory assist
PEEP: Positive end-expiratory pressure
PSV: Pressure support ventilation
ROI: Regions of interest
Vt: Tidal volume
